# All-Optical Terahertz Dual-Band Logic Gates Based on Unidirectional Modes

**DOI:** 10.3390/mi17050509

**Published:** 2026-04-22

**Authors:** Dewang Guo, Yun You, Zhimin Liu, Jie Xu

**Affiliations:** 1 School of Science, East China Jiaotong University, Nanchang 330013, China; 2 School of Medical Information and Engineering, Southwest Medical University, Luzhou 646000, China

**Keywords:** logic gates, surface magnetoplasmons (SMPs), unidirectional propagation, nonlocal effects

## Abstract

All-optical logic gates have emerged as a critical technology for enabling broadband, low-loss, and high-speed communication systems, addressing the inherent bandwidth limitations of electronic counterparts. Here, we propose a Y-shaped structure leveraging unidirectional modes in the terahertz regime, which enables the realization of multifunctional all-optical logic gates within the lower- and upper-frequency bandwidth regions, including, but not limited to, AND, OR, NOT, and XNOR gates. Numerical simulations and theoretical analyses confirm that the proposed logic gates exhibit robust one-way propagation characteristics, with electromagnetic signals demonstrating complete immunity to backscattering even in the presence of structural defects. Furthermore, nonlocal effects are found to have a negligible impact on the operational bandwidths of our design. Building upon this Y-shaped configuration, we further develop an all-optical digital logic system (AODLS) capable of supporting bifrequency multi-input and multi-output logic operations. When lower- and upper-frequency signals are injected into separate input ports, their corresponding output signals remain fully independent, eliminating cross-talk and enabling true parallel computation. This dual-band parallel processing capability represents a significant advance over conventional single-band all-optical logic systems, opening new avenues for high-throughput all-optical computing and integrated photonic circuits.

## 1. Introduction

Since the transistor was first invented in 1947, there has been great developments in electronic technology. However, affected by the constraints of both Moore’s law and Joule’s law, electronic communications suffer from defects such as cross-talk [1,2]. All-optical communications, owing to their ultra-fast data processing, error-free transmission [3], and minimal or even zero energy loss [4], reveal their potential as candidates for next-generation communication technology.

All-optical logic gates, which fulfill various logical functions, have received much attention for their potential applications in all-optical computing systems [5,6,7] and photonic integrated circuits [8,9]. Nowadays, there are several methods to realize all-optical logic functions, such as semiconductor optical amplifier (SOA) [10,11], optical interference effect [12,13], third-order nonlinear effect [14,15,16], and interferometry [17,18,19]. In fact, due to the unavoidable reflections in conventional optical logic gates, the contrast ratios (CRs) of most logic gates are typically below 30 dB. In numerous investigations focusing on sub-wavelength all-optical logic gates, the conventional surface magnetoplasmons (SMPs) fail to be strictly unidirectional, and there will always be propagating modes in both directions once nonlocal effects are considered [20]. Note that in ref. [21], while in certain frequency regimes SMPs modes clearly lose their unidirectionality owing to nonlocal effects, spatial dispersion does not always prevent the existence of unidirectional surface waves. Indeed, it can be shown that the upper bulk mode bandgap (opened due to time-reversal-symmetry breaking) is characterized by a nonzero topological invariant number; topologically protected unidirectional surface waves can emerge when an interface is formed with a material having a bulk mode bandgap with different topological properties. In addition, while the conventional SMPs are not strictly unidirectional in the lower bulk mode bandgap, in most cases of interest they may be practically unidirectional due to the unavoidable losses that would largely attenuate the backward propagating modes. The one-way electromagnetic (EM) modes are theoretically topologically protected, which has been theoretically demonstrated [22,23] and experimentally proven [24,25] by many groups. Notably, in recent microwave-related research based on unidirectional waveguides, they have successfully implemented AND and OR logic operations under a unified logic-level definition, which is more suitable for cascading applications [26].

In this paper, we propose a robust AODLS based on unidirectional modes in the terahertz regime. The Y-shaped module of the AODLS enables the implementation of fundamental logic gates across distinct lower- and upper-frequency bands, including, AND, OR, NOT, and XNOR gates. Since nonlocality-immune SMPs modes are unidirectional and immune to backscattering, this implies an ultra-high contrast ratio. Most importantly, the AODLS supports bifrequency multi-input multi-output logic operations, where upper- and lower-frequency signals can be processed simultaneously with signal isolation between input and output ports. This intrinsic signal decoupling enables true parallel computation, a capability that surpasses the performance of single-band all-optical logic systems reported in the existing literature. This work establishes a new paradigm for all-optical calculations, offering a scalable solution for the development of high-throughput integrated photonic circuits and next-generation optical information processing systems.

## 2. Physical Model

**Figure 1:**
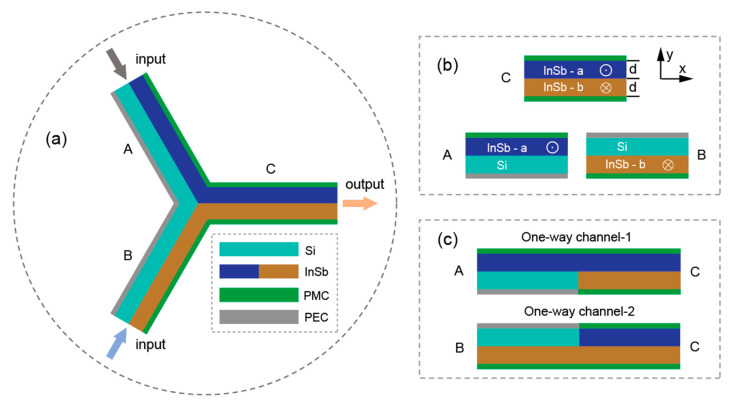
(**a**) The schematic of the Y-shaped structure of all-optical logic operations. (**b**) The three types of arms. (**c**) Pre-designed two one-way channels. Note that we use *ω_c_^a^*, *ω_c_^b^* to clarify the procession *ω_c_* for blue-colored InSb-a, and brown-colored InSb-b layers, respectively.

The Y-shaped structure is a widely adopted physical model in the field of all-optical logic devices; it has been extensively studied over the past few decades [27,28,29,30]. As schematically illustrated in Figure 1, the proposed structure consists of three symmetric straight arms (labeled ‘A’, ‘B’, and ‘C’), each constructed from alternating layers of dielectric (Si) and semiconductor (InSb), with both layers having a uniform thickness *d*. The Si layer is bounded by a perfect electric conductor (PEC), while the InSb layer is terminated by a perfect magnetic conductor (PMC). Notably, the InSb segments are divided into two regions (InSb-a and InSb-b) with distinct magnetic configurations. The blue-colored InSb-a is magnetized along the +*z* direction by an external magnetic field *B*_0_, whereas the brown-colored InSb-b is magnetized along the -*z* direction. The equivalent magnetic bias configurations could potentially be realized through engineered current-driven electromagnetic fields (patterned current arrays) to generate spatially varying effective magnetic fields with opposite orientations. The *z*-direction thickness of the InSb layers is treated as infinitely extended in the two-dimensional model, with all results readily extending to three-dimensional structures by adjusting the *z*-dimension boundary conditions. To implement basic logical operations based on unidirectional modes, the key lies in establishing two independent unidirectional channels to achieve efficient EM wave transmission.

In our work, the relative permittivity of InSb-a, and InSb-b, influenced by the magnetic field, are given by:

(1) $${\overset{↔}{\varepsilon }}_{a}=\left[\begin{array}{ccc} \varepsilon_{1}^{a} & i\varepsilon_{2}^{a} & 0\\ -i\varepsilon_{2}^{a} & \varepsilon_{1}^{a} & 0\\ 0 & 0 & \varepsilon_{3}^{a} \end{array}\right],\;\;\;\;\;{\overset{↔}{\varepsilon }}_{b}=\left[\begin{array}{ccc} \varepsilon_{1}^{b} & -i\varepsilon_{2}^{b} & 0\\ i\varepsilon_{2}^{b} & \varepsilon_{1}^{b} & 0\\ 0 & 0 & \varepsilon_{3}^{b} \end{array}\right]$$

with

(2) $$\varepsilon_{1}=\varepsilon_{\infty }(1-\frac{(\omega +i\nu ){\omega_{p}}^{2}}{\omega [{\left(\omega +i\nu \right)}^{2}-{\omega_{c}}^{2}]}),\;\;\;\;\varepsilon_{2}=\varepsilon_{\infty }\frac{\omega_{c}{\omega_{p}}^{2}}{\omega [{\left(\omega +i\nu \right)}^{2}-{\omega_{c}}^{2}]},\;\;\;\;\varepsilon_{3}=\varepsilon_{\infty }(1-\frac{{\omega_{p}}^{2}}{\omega (\omega +i\nu )})$$

where *ε*_∞_ denotes the high-frequency permittivity of the semiconductor, *ω_p_* is the plasma frequency of the semiconductor, *ω* is the angular frequency, and *ω_c_* = *eB*_0_/m* (*e* and m* represent the charge and effective mass of the electron) is the electron cyclotron frequency. In this paper, *ε*_∞_ = 15.6 (InSb), *ε_d_* = 11.68 (Si). In the following analytical derivation, the semiconductor is assumed to be lossless for clarity, while the realistic material loss will be incorporated into subsequent full-wave transmission simulations. To establish two independent unidirectional channels for efficient EM signal transmission, it is essential to characterize the dispersion relation of the SMPs in those arms. According to Maxwell’s equations with appropriate boundary conditions, one may readily calculate the dispersion equations in the above three arms using:

(3) $$\frac{\varepsilon_{2}^{a}}{\varepsilon_{1}^{a}}k+\frac{\alpha_{a}}{\tanh(\alpha_{a}d)}+\frac{\varepsilon_{v}^{a}}{\varepsilon_{d}}\alpha_{d}\tanh(\alpha_{d}d)=0,\;\;\;\;\;\;\;(‘A’)$$

(4) $$\frac{\varepsilon_{2}^{b}}{\varepsilon_{1}^{b}}k+\frac{\alpha_{b}}{\tanh(\alpha_{b}d)}+\frac{\varepsilon_{v}^{b}}{\varepsilon_{d}}\alpha_{d}\tanh(\alpha_{d}d)=0,\;\;\;\;\;\;\;(‘B’)$$

(5) $$\varepsilon_{v}^{b}\left[\frac{\varepsilon_{2}^{a}}{\varepsilon_{1}^{a}}k+\frac{\alpha_{a}}{\tanh(\alpha_{a}d)}\right]+\varepsilon_{v}^{a}\left[\frac{\varepsilon_{2}^{b}}{\varepsilon_{1}^{b}}k+\frac{\alpha_{b}}{\tanh(\alpha_{b}d)}\right]=0,\;\;\;\;\;\;\;(‘C’)$$

where *α_a_* = $\sqrt{k^{2}-\varepsilon_{v}^{a}{{\;k}_{0}}^{2}}$, *α_b_* = $\sqrt{k^{2}-\varepsilon_{v}^{b}{{\;k}_{0}}^{2}}$, and *α_d_* = $\sqrt{k^{2}-\varepsilon_{d}{{\;k}_{0}}^{2}}$ respectively represent the transverse attenuation coefficients of the SMPs in the InSb-a, InSb-b, and Si layers. From the dispersion equations, it is evident that the SMPs in the arms exhibit contrasting propagation properties for opposite wavenumbers, reflecting the nonreciprocity effect. Crucially, tuning the external magnetic field enables the creation of a unidirectional propagation region, where EM waves are confined to travel exclusively along a single direction. With the dispersion equation of the SMPs derived, the asymptotic frequencies (AFs) can be readily calculated. These AFs define the boundaries of the complete one-way propagation (COWP) bands, within which we observe that:

(6) $$\omega_{sp}^{\left(+\right)}=\left\{\begin{array}{c} \omega_{sp}^{\left(+1\right)}=\omega_{a}=\frac{1}{2}(\sqrt{{\omega_{c}}^{2}+4{\omega_{p}}^{2}}-\omega_{c})\\ \omega_{sp}^{\left(+2\right)}=\frac{1}{2}(\sqrt{{\omega_{c}}^{2}+4{\omega_{p}}^{2}\frac{\varepsilon_{\infty }}{\varepsilon_{\infty }+\varepsilon_{d}}}+\omega_{c}) \end{array}\right.$$

(7) $$\omega_{sp}^{\left(-\right)}=\left\{\begin{array}{c} \omega_{sp}^{\left(-1\right)}=\frac{1}{2}(\sqrt{{\omega_{c}}^{2}+4{\omega_{p}}^{2}\frac{\varepsilon_{\infty }}{\varepsilon_{\infty }+\varepsilon_{d}}}-\omega_{c})\\ \omega_{sp}^{\left(-2\right)}=\omega_{b}=\frac{1}{2}(\sqrt{{\omega_{c}}^{2}+4{\omega_{p}}^{2}}+\omega_{c}) \end{array}\right.$$

for arms ‘A’/‘B’. *ω_sp_*^(+)^ and *ω_sp_*^(−)^ indicate the AFs in the limits of *k* → +∞ and *k* → −∞, respectively. In fact, the values of *ω_a_* and *ω_b_* correspond to the zero point of *ε_v_*. And

(8) $$\omega_{sp}^{\left(+\right)}=\left\{\begin{array}{c} \omega_{sp}^{\left(+1\right)}=\omega_{c}\\ \omega_{sp}^{\left(+2\right)}=\omega_{a}=\frac{1}{2}(\sqrt{{\omega_{c}}^{2}+4{\omega_{p}}^{2}}-\omega_{c}) \end{array}\right.$$

(9) $$\omega_{sp}^{\left(-\right)}=\omega_{b}=\frac{1}{2}(\sqrt{{\omega_{c}}^{2}+4{\omega_{p}}^{2}}+\omega_{c})$$

for ‘C’, where the cyclotron frequencies of InSb-a and InSb-b are equal (*ω_c_^a^* = *ω_c_^b^*), analyses of Equations (6)–(9) reveal that the three arms share identical AFs, denoted as *ω_a_* and *ω_b_*, significantly simplifying the design process for effective broadband.

**Figure 2:**
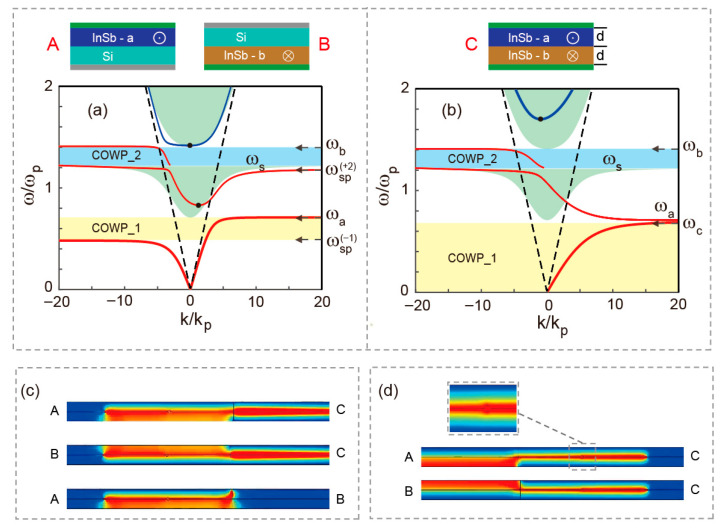
(**a**) The dispersion diagrams of arms ‘A’/‘B’, *ω_c_^a^* = *ω_c_^b^* = 0.7*ω_p_* and *d* = 0.05*λ_p_*. (**b**) The dispersion diagrams of arm ‘C’, *ω_c_^a^* = *ω_c_^b^* = 0.7*ω_p_* and *d* = 0.05*λ_p_*. (**c**,**d**) Electromagnetic signal transmission through channels operating at 0.6*f_p_* and 1.3*f_p_*, respectively.

The implementation of broadband all-optical logic operations based on unidirectional SMPs modes necessitates the establishment of independent one-way propagation channels and sufficiently broad COWP bands. We choose *ω_c_^a^* = *ω_c_^b^* = 0.7*ω_p_* (*ω_p_* = 4π × 10^12^ rad/s), *d* = 0.05*λ_p_* (*λ_p_* = 2π*c*/*ω_p_*) in the following work. Under this parameter configuration, the dispersion curves for the SMPs in the arms ‘A’/‘B’ and ‘C’ are calculated and plotted in Figure 2a and Figure 2b, respectively. In these figures, the red solid lines represent the dispersion curves of the SMPs modes, while the blue solid curves correspond to the lowest-order bulk modes within the green-shaded bulk zones, and the black dashed lines indicate light-lines in the medium. Black horizontal arrows mark the values of AFs, and black dots denote cutoff frequencies *ω_cf_*, which are influenced by the dielectric thickness *d*. Based on our calculations, the bandwidths of the COWP bands in the arms ‘A’/‘B’ are 0.48*ω_p_* < *ω* < 0.71*ω_p_* (COWP_1), 1.22*ω_p_* < *ω* < 1.41*ω_p_* (COWP_2). For arm ‘C’, the COWP band bandwidths are 0 < *ω* < 0.7*ω_p_* (COWP_1), 1.22*ω_p_* < *ω* < 1.41*ω_p_* (COWP_2). Consequently, two distinct unidirectional propagation channels are established (‘A → C’ and ‘B → C’) in the lower one-way regions (0.48*ω_p_* < *ω* < 0.7*ω_p_*). Conversely, the EM signal transmission from arm ‘A’ to arm ‘B’ is effectively suppressed, demonstrating the non-reciprocal characteristic essential for optical logic isolation. Here, the propagation direction is reversed in the upper one-way regions (1.22*ω_p_* < *ω* < 1.41*ω_p_*), enabling two one-way channels (‘C → A’ and ‘C → B’), while the signal transmission between arms ‘A’ and ‘B’ remains prohibited. Actually, the designed bandwidths are sufficiently broad to ensure operational robustness and facilitate practical device implementation.

To verify the theoretical predictions regarding the unidirectional propagation characteristics of SMPs and the inter-arm coupling effects, full-wave EM simulations were conducted using finite element method (FEM). The simulation setup, as illustrated in Figure 2c,d, incorporates structural perturbations in the form of air holes (radius *r* = 1.5 um) placed at the InSb-InSb interfaces with *ν* = 0.002*ω_p_*, and SMPs are excited using a magnetic current source. We chose two frequencies *f* = 0.6*f_p_* and *f* = 1.3*f_p_*, corresponding to the lower and upper unidirectional frequency bands of interest. The simulated magnetic field distributions confirm the robust one-way channeling behavior. As shown in Figure 2c, the EM signal is efficiently guided through the unidirectional channels ‘A → C’ and ‘B → C’, while the propagation from arm ‘A’ to arm ‘B’ is effectively suppressed at their interface for *f* = 0.6*f_p_*. This observation validates the predicted lower-band isolation. Similarly, Figure 2d demonstrates that at *f* = 1.3*f_p_*, the EM signal propagates exclusively from arm ‘C’ to arms ‘A’ or ‘B’, with no signal transfer occurring between arms ‘A’ and ‘B’. These results are in excellent agreement with the theoretical dispersion analysis. In addition, the EM signal is observed to circumvent the introduced air-hole defects entirely, with the defects exerting a negligible influence on the transmission characteristics of the one-way SMPs, further demonstrating the remarkable robustness of the unidirectional propagation against structural imperfections. The proposed design exhibits robust tolerance to material defects and realistic fabrication tolerances, thereby verifying its practical feasibility for real-world implementation.

**Figure 3:**
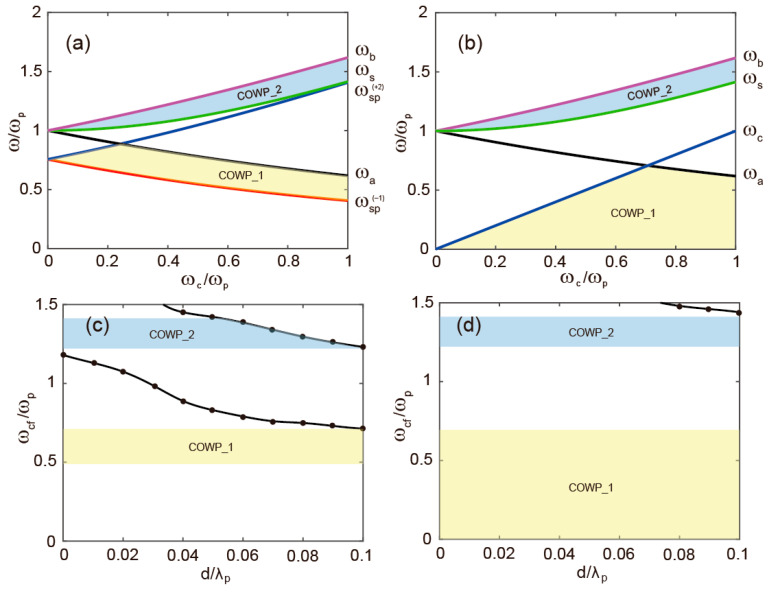
(**a**) The asymptotic frequencies (AFs) are plotted as functions of *ω_c_* in the arms ‘A’/‘B’. (**b**) AFs are plotted as functions of *ω_c_* in the arm ‘C’. (**c**,**d**) The cutoff frequencies *ω_cf_* as functions of the thickness *d* in the arms ‘A’/‘B’, ‘C’, respectively.

The bandwidths of the unidirectional propagation region in the three arms can be effectively tuned by adjusting the external magnetic field (*B*_0_). To systematically investigate the influence of the magneto-static field on the COWP bands, we plot the AFs as functions of the cyclotron frequency *ω_c_* for *d* = 0.05*λ_p_*. As shown in Figure 3a, for arms ‘A’/‘B’, increasing *B*_0_ initially broadens and subsequently narrows the COWP_1 band, while the COWP_2 band exhibits a monotonic expansion in bandwidth. In contrast, Figure 3b reveals more complex variations in the COWP bands for arm ‘C’, with the maximum total bandwidth achieved at *ω_c_* = 0.7*ω_p_*.

In addition to the magnetic field, the dielectric layer thickness *d* also plays a critical role in shaping the COWP bands, as the excitation of reverse-propagating SMPs can reduce the effective unidirectional window. The relationship between *d* and *ω_cf_* at *ω_c_* = 0.7*ω_p_* is examined in Figure 3c,d. The results indicate that for *d* < 0.053*λ_p_*, the COWP bands in arms ‘A’/‘B’ and ‘C’ remain largely unaffected. However, once *d* > 0.053*λ_p_*, the upper-frequency COWP_2 band in arms ‘A’/‘B’ undergo significant narrowing. Therefore, to ensure adequate operational bandwidths in both the lower- and upper-frequency regimes while maintaining strong unidirectional confinement, we select the optimal parameter of *d* = 0.05*λ_p_* and *ω_c_* = 0.7*ω_p_* for the subsequent design and analysis of the proposed all-optical logic device.

## 3. Realization of Basic Logic Gates

**Figure 4:**
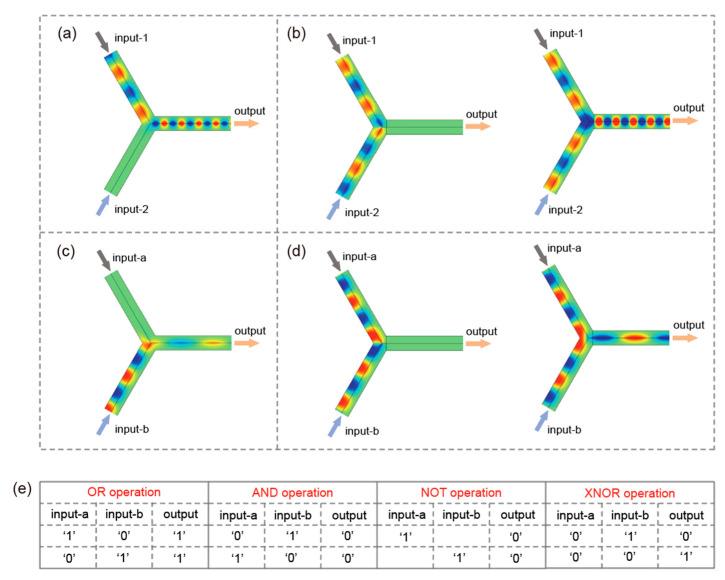
(**a**) Simulated field distributions of the Y-shaped structure at *f* = 0.6*f_p_*, with EM signal excitation applied to a single input port. (**b**) Two input ports are excited with EM signals of phases 3*pi/2 and pi/2 in the left figure, and both input ports are driven by signals with the phase of 3*pi/2 in the right figure. (**c**,**d**) The external magnetic field direction applied to the InSb-a and InSb-b layers are reversed, with simulated field distributions at *f* = 1.3*f_p_*. (**e**) The truth tables of the OR, AND, NOT, and XNOR operations.

As discussed in the context of Figure 2, the arms ‘A’, ‘B’, and ‘C’ support unidirectional forward propagation modes within the operational frequency range of 0.48*ω_p_* < *ω* < 0.7*ω_p_*. Leveraging the two distinct one-way channels established in this frequency band, the proposed Y-shaped structure can function as a platform for all-optical logic gates, including, but not limited to, AND, OR, NOT, and XNOR gates, as schematically illustrated in Figure 4a,b. In the logic operation scheme, arms ‘A’ and ‘B’ serve as input ports, while arm ‘C’ acts as the output port. Figure 4a presents simulated field distributions for the Y-shaped structure at *f* = 0.6*f_p_* when the EM signal is excited in only one of the input ports. For the OR gate implementation under positive logic convention, the presence of an EM signal in an arm is defined as logic ‘1’, whereas the absence of signal (zero-energy state) corresponds to logic ‘0’. Accordingly, when any input port receives a logic ‘1’ (unidirectional EM signal), the output port also exhibits logic ‘1’, thereby realizing the OR gate function. Specifically, for input combinations ‘10’ or ‘01’, the output power reaches 0.426 mW with a transmittance of 0.316, corresponding to logic state ‘1’; for input ‘11’, the output power increases to 1.7 mW with a transmittance of 0.63, also corresponding to logic state ‘1’; and for input ‘00’, no output power (0 mW) or transmittance (0) is detected, corresponding to logic state ‘0’. In contrast, the AND gate operation requires the adoption of a negative logic scheme. Here, the propagation of EM signal is interpreted as logic ‘0’, while the absence of energy flow is regarded as logic ‘1’. Under this principle, if any input port is excited with EM signal (logic ‘0’), the output will correspondingly yield logic ‘0’. This logical inversion, combined with the unidirectional transmission characteristics of the structure, enables the realization of the AND gate within the same physical layout.

Similarly, the NOT gate operation is implemented using the negative logic scheme, wherein the presence of EM energy at the input port is defined as logic ‘1’, while the output port yields logic ‘0’. Under this convention, the input logic ‘1’ is converted to the output logic ‘0’, thereby realizing the logical inversion characteristic of the NOT gate. Furthermore, the XNOR gate can be achieved by injecting EM signals with distinct phases into the two input ports. In this scheme, the signal phase of pi/2 is assigned to logic ‘1’, and the phase of 3*pi/2 corresponds to logic ‘0’. Two representative simulations are performed on the Y-shaped structure, as shown in Figure 4b. In the left figure, when the two input ports are excited with EM signals of phases 3*pi/2 and pi/2, respectively, the zero-energy state is observed at the output port. In the right figure, when both input ports are driven by signals with the phase of 3*pi/2, the EM energy couples unidirectionally into arm ‘C’, resulting in a clear output signal. Notably, input combinations ‘10’ or ‘01’ yield an output power of 1.04 × 10^−7^ mW with a transmittance of 7.7 × 10^−8^, representing logic ‘0’, while inputs ‘11’ or ‘00’ result in an output power of 1.7073 mW with a transmittance of 0.63, corresponding to logic ‘1’. The output characteristics of the unidirectional SMPs are highly dependent on the applied magnetic field, which enables tolerance to phase deviations via fine-tuning. The corresponding truth tables for the OR, AND, NOT, and XNOR operations are summarized in Figure 4e. All simulation results align closely with the theoretical predictions, demonstrating the reliable logic-level response of the proposed structure. Accordingly, the Y-shaped module is capable of performing multiple logic operations and acts as a multifunction logic gate, which has potential for high-performance optical communication or calculation.

Notably, the arms ‘A’, ‘B’, and ‘C’ also support unidirectional propagation modes within the upper-frequency operating band of 1.22*ω_p_* < *ω* < 1.41*ω_p_*, where the EM energy propagates in the backward direction. To implement logic operations in this frequency regime, the external magnetic field direction applied to the InSb-a and InSb-b layers can be reversed, thereby inverting the propagation direction in the three arms. Following the analogous analysis that presents in Figure 4a,b, the OR, AND, NOT, and XNOR logic functions are successfully demonstrated in Figure 4c,d when *f* = 1.3*f_p_*. In the OR gate, for inputs ‘10’ or ‘01’, the output power is 0.475 mW with a transmittance of 0.416, representing logic ‘1’; for input ‘11’, the output power rises to 1.9 mW with a transmittance of 0.832, representing logic ‘1’; and input ‘00’ produces no detectable output power or transmittance, representing logic ‘0’. In the XNOR gate, inputs ‘10’ or ‘01’ result in 1.35 × 10^−10^ mW output power and 1.18 × 10^−10^ transmittance, corresponding to logic ‘0’, while inputs ‘11’ or ‘00’ yield an output power of 1.9 mW with a transmittance of 0.832, corresponding to logic ‘1’. The operational principle of these gates relies exclusively on stable unidirectional EM signals, which afford significantly higher discrimination precision compared to alternative approaches. For example, in the simulated OR gate operation, the logic ‘1’ state corresponds to a clearly defined EM signal, whereas the logic ‘0’ state exhibits no detectable energy, resulting in a sharply distinct contrast. A distinctive feature of this work is that the SMPs-based logic gates can operate within two continuous and relatively broad complete COWP bands. The dual-band capability enables flexible, frequency-selective logic functionality without altering the physical geometry, thereby enhancing the adaptability of the device for programmable photonic circuits.

**Figure 5:**
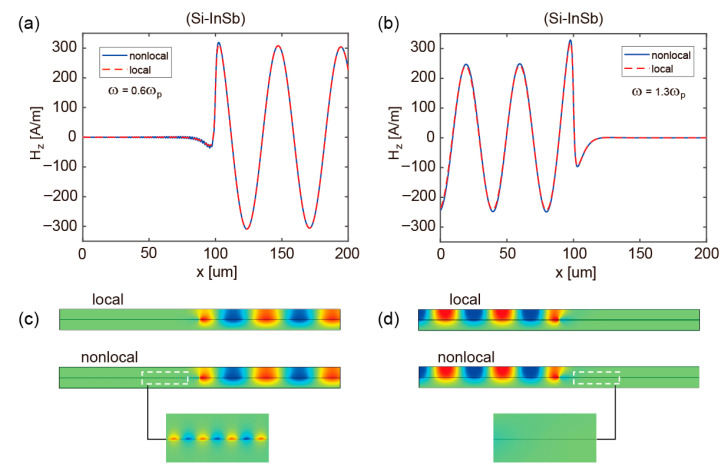
(**a**,**b**) The one-dimensional magnetic-field profiles of Si-InSb structure at 0.6*f_p_* and 1.3*f_p_*, respectively. (**c**,**d**) Corresponding magnetic-field distributions of the Si-InSb structure under local and nonlocal models at 0.6*f_p_* and 1.3*f_p_*.

**Figure 6:**
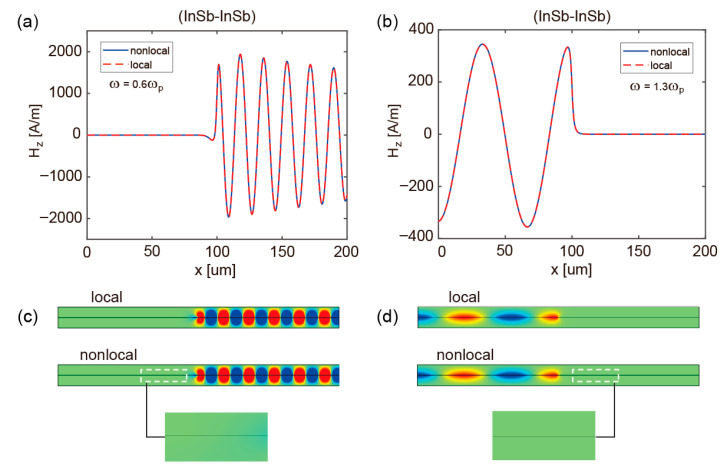
(**a**,**b**) The one-dimensional magnetic-field profiles of InSb-InSb structure at 0.6*f_p_* and 1.3*f_p_*, respectively. (**c**,**d**) Corresponding magnetic-field distributions of the InSb-InSb structure under local and nonlocal models at 0.6*f_p_* and 1.3*f_p_*.

To evaluate the fundamental impact of nonlocal effects on the operational integrity of the proposed all-optical logic gates, a comparative analysis of the pattern distributions in arms ‘A’/‘B’ and ‘C’ is conducted under both local and nonlocal models at *f* = 0.6*f_p_* and 1.3*f_p_*, as shown in Figure 5 and Figure 6. These findings collectively reveal the pronounced robustness of both Si-InSb and InSb-InSb structures against nonlocal perturbations. For the Si-InSb configuration, nonlocal interactions induce a weak excitation of reverse-propagating SMPs at 0.6*f_p_* in Figure 5a, with only minor deviations from the one-dimensional magnetic field distribution predicted by the local model. This indicates that the core unidirectional transmission property of SMPs remains effectively preserved for practical device applications. In contrast, Figure 5b reveals that the Si-InSb structure demonstrates complete immunity to nonlocal effects at 1.3*f_p_*, sustaining topologically protected unidirectional surface waves without any observable degradation in propagation direction. The InSb-InSb structure displays even stronger robustness, as illustrated in Figure 6. At both the lower (*f* = 0.6*f_p_*) and upper (*f* = 1.3*f_p_*) operational frequencies, the field distribution curves predicted by the nonlocal model show perfect consistency with those from the local model. The perfect agreement confirms the broadband immunity of the InSb-InSb structure to nonlocal effects, underscoring its exceptional stability for sustaining unidirectional transmission modes. These findings demonstrate that nonlocal effects neither compromise the established unidirectional channels nor degrade the computational performance of all-optical logic gates.

## 4. The AODLS for Multi-Input Logic Operations

**Figure 7:**
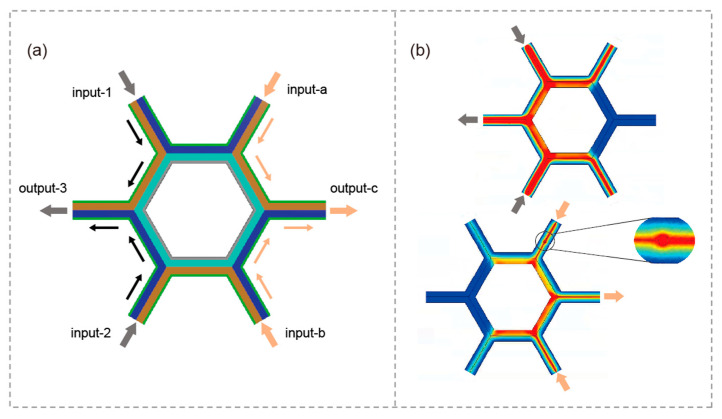
(**a**) The schematic of AODLS structure. (**b**) Transmission of EM wave in AODLS.

Optical computations are characterized by super-fast data transmissions relying on fan-in and fan-out, and our proposed unidirectional-modes-based logic gates have two broad operating bands. Building on this foundational capability, we now present a systematic design approach for integrating this module into AODLS. The schematic diagram of the proposed all-optical AODLS is presented in Figure 7a. To clearly illustrate the signal flow, arrows have been incorporated into the schematic, indicating the propagation directions within the unidirectional channels. The system architecture supports four distinct input configurations for two independent output ports, enabling versatile logic operations. At the lower-frequency band operations (0.48*f_p_* < *f* < 0.7*f_p_*), the EM signals are injected into the ‘input-1’ and ‘input-2’ ports. The corresponding output signal is collected from the ‘output-3’ port. When operating in this upper-frequency band (1.22*f_p_* < *f* < 1.41*f_p_*), the input signals are directed into the ‘input-a’ and ‘input-b’ ports, with the output retrieved from the ‘output-c’ port.

To experimentally validate the functionality and robustness of the proposed AODLS, full-wave EM simulations were performed using FEM, with the schematic and simulated field distributions presented in Figure 7b. Imperfections on the InSb-InSb interface are introduced as air holes with *r* = 1.5 um to demonstrate the robust unidirectionality of the incident EM modes. The input EM wave perfectly bypasses the introduced air hole defects at the input port and subsequently splits into two guided waves. This confirms the robustness of the topological one-way channels against backscattering and localization induced by interface disorders. Notably, virtually no inter-channel cross-talk or spurious noise is observed during the entire logic operation process. The input/output propagation characteristics align perfectly with the theoretical predictions based on the dispersion analysis. Most importantly, inputs applied at ‘input-1’ and ‘input-2’ have no measurable effect on ‘output-c’. Similarly, signals injected into ‘input-a’ and ‘input-b’ do not influence ‘output-3’, allowing for concurrent operation. Hence, the signal inputs and outputs are independent, which is promising for parallel computation. Surely, the OR, AND, NOT, and XNOR logical operations are also implemented in AODLS. It is worth mentioning that the AODLS works with two outputs and could be easily extended to the four-output logic device by suitably assembling the Y-shaped modules at the output ports. The AODLS shows significant promise in complex logic operations due to its expansibility, precision, and broadband characteristics. Compared to conventional electronic logic gates, which rely on distinguishing between relatively high and low voltage levels, the unidirectional all-optical logic gates offer a distinct advantage, given that the logical state is defined by the presence or absence of the EM signal.

## 5. Conclusions

In this work, we have proposed a novel, robust, AODLS operating in the terahertz regime. Theoretical analysis and full-wave simulations demonstrate that the Y-shaped module of the AODLS can independently implement fundamental logic functions (not limited to, AND, OR, NOT, and XNOR gates) within the lower- and upper-frequency regions. The lower- and upper-frequency bands are defined by 0.48*ω_p_* < *ω* < 0.7*ω_p_* and 1.22*ω_p_* < *ω* < 1.41*ω_p_*, respectively. The unidirectional propagation characteristics remain robust against structural imperfections and operational bandwidths are completely unaffected by nonlocal effects, ensuring operational stability. In addition, by connecting Y-shaped modules at the output ports, the AODLS can be extended to the four-output logic device. The upper-frequency and lower-frequency operational modes are entirely independent, with no cross-talk observed on their output ports. This intrinsic port isolation enables genuine parallel computation on a single integrated platform. Owing to the broad operating band, error-free transmission and ultra-fast data processing, our proposed AODLS could become a novel platform for optical calculations and optical communication.

## Data Availability

The original contributions presented in this study are included in the article. Further inquiries can be directed to the corresponding authors.
